# Adaptive cellular response of the *substantia nigra* dopaminergic neurons upon age‐dependent iron accumulation

**DOI:** 10.1111/acel.13694

**Published:** 2022-08-19

**Authors:** Kujin Kwon, Hwapyeong Cho, Soyeon Lee, Eun Jeong Cho, Weonjin Yu, Catherine Yen Li Kok, Hyunsoo Shawn Je, Jae‐Ick Kim, Hyung Joon Cho, Taejoon Kwon

**Affiliations:** ^1^ Department of Biomedical Engineering, College of Information and Biotechnology Ulsan National Institute of Science and Technology (UNIST) Ulsan Korea; ^2^ Department of Biological Sciences, College of Information and Biotechnology Ulsan National Institute of Science and Technology (UNIST) Ulsan Korea; ^3^ Neuroscience and Behavioral Disorders Program Duke‐National University of Singapore (NUS) Medical School Singapore City Singapore; ^4^ Advanced Bioimaging Center, Academia Singapore City Singapore; ^5^ Center for Genome Integrity Institute for Basic Science (IBS) Ulsan Korea

**Keywords:** aging, iron accumulation, iron toxicity, magnetic resonance imaging, *substantia nigra*, transcriptome

## Abstract

Progressive iron accumulation in the *substantia nigra* in the aged human brain is a major risk factor for Parkinson's disease and other neurodegenerative diseases. Heavy metals, such as iron, produce reactive oxygen species and consequently oxidative stress in cells. It is unclear, however, how neurons in the *substantia nigra* are protected against the age‐related, excessive accumulation of iron. In this study, we examined the cellular response of the *substantia nigra* against age‐related iron accumulation in rats of different ages. Magnetic resonance imaging confirmed the presence of iron in 6‐month‐old rats; in 15‐month‐old rats, iron accumulation significantly increased, particularly in the midbrain. Transcriptome analysis of the region, in which iron deposition was observed, revealed an increase in stress response genes in older animals. To identify the genes related to the cellular response to iron, independent of neurodevelopment, we exposed the neuroblastoma cell line SH‐SY5Y to a similar quantity of iron and then analyzed their transcriptomic responses. Among various stress response pathways altered by iron overloading in the rat brain and SH‐SY5Y cells, the genes associated with topologically incorrect protein responses were significantly upregulated. Knockdown of HERPUD1 and CLU in this pathway increased susceptibility to iron‐induced cellular stress, thus demonstrating their roles in preventing iron overload‐induced toxicity. The current study details the neuronal response to excessive iron accumulation, which is associated with age‐related neurodegenerative diseases.

## Graphical abstract

Iron is regionally accumulated in the aged brain, but how neuronal cells can be tolerated in this stressed condition is not fully elucidated. Here, the *substantia nigra* of aged rat brains was examined for iron accumulation with the MRI and their cellular responses with transcriptome analysis. Our study showed that CLU and HERPUD1, two genes involved in the unfolded protein response in the ER, have crucial roles in protecting cells from iron toxicity.

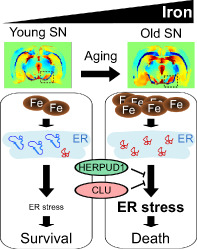

**Abbreviations**
AD: Alzheimer's disease
DEG: differentially expressed gene
ER: endoplasmic reticulum
GSEA: gene set enrichment analysis
MRI: magnetic resonance imaging
NM: neuromelanin
PD: Parkinson's disease
QSM: quantitative susceptibility mapping
SN: *substantia nigra*

## 1 INTRODUCTION

Iron is critical to biological processes in the central nervous system, such as myelin production, and neurotransmitter synthesis and metabolism (Friedrich et al., 2021). An imbalance in iron homeostasis leads to the generation of reactive oxygen species through Fenton's reaction, which damages DNA and induces neuronal cell death, including iron‐dependent cell death (ferroptosis; Ward et al., 2014). Iron, however, accumulates progressively in the brain throughout life, particularly in the *substantia nigra* (SN) and *globus pallidus* (Zecca et al., 2004). Ferritin and neuromelanin, furthermore, chelate iron and protect neurons against iron toxicity (Zecca et al., 2001). When iron levels, however, exceed the iron buffering and sequestering capacity of the cell, a high concentration of iron in the labile iron pool induces oxidative damage‐induced cell death, leading to neurodegenerative diseases (Ward et al., 2014).

Abnormally high iron concentrations accumulate in the *hippocampus* in Alzheimer's disease (AD) (Bartzokis, 2004) and the *SN* in Parkinson's disease (PD) (Dexter et al., 1991; Riederer et al., 1989; Wallis et al., 2008). The increase in brain iron levels with age and the high quantity of iron found in the *SN* of PD patients suggest that iron dyshomeostasis may occur during healthy aging, which leads to neurodegeneration (Zucca et al., 2017). During the development of PD, iron levels increase in several brain regions; however, substantial neuronal loss in the early stage of the disease is restricted to the *SN*, suggesting that additional biological factors associated with a pathological characteristic of iron (Wang et al., 2016). Toxic, iron‐catalyzed, dopamine byproducts are produced in the cytoplasm of dopaminergic neurons with high iron concentrations (Exner et al., 2012). Moreover, iron or dopamine oxidation derivatives promote pathological alpha‐synuclein oligomerization, causing the loss of dopaminergic neurons (Minakaki et al., 2020) in PD. The *SN*, therefore, is vulnerable to neurodegeneration via iron accumulation.

The genetic mechanisms associated with age‐related iron accumulation in the *SN* have been partially elucidated. Knocking out Nrf2, for example, reduces iron accumulation in the *SN* of aging mice (Han et al., 2021). Furthermore, age‐dependent miR‐29 upregulation limits iron uptake in the killifish model (Ripa et al., 2017). Little is known, however, about the transcriptome landscape and its underlying mechanisms that counteract age‐dependent iron accumulation in the *SN*.

In this study, we examined the expression profile of age‐related iron accumulation in the *SN* of rats of different ages. Magnetic resonance imaging (MRI) revealed significantly elevated iron levels in the *SN* of 15‐month‐old rats. Analysis of differentially expressed genes showed that the expression of apoptosis‐related genes increased in older animals. To precisely determine the genes related to the iron response, independent of neurodevelopment, we challenged the human neuroblastoma SH‐SY5Y cell line with ferrous irons and then analyzed their transcriptomic responses. Among the various stress responses altered by iron overload in the rat brain and SH‐SY5Y cells, the endoplasmic reticulum (ER) stress‐related pathway linked to incorrect protein folding was significantly enriched. We further characterized HERPUD1 and CLU associated with this pathway; their downregulation increased susceptibility to stress following exposure to high doses of iron. Our study details the neuronal response to excessive iron accumulation, which is associated with age‐related neurodegenerative diseases.

## 2 MATERIALS AND METHODS

### 2.1 Animal preparation

Fifteen‐month‐old and 6‐month‐old Sprague Dawley male rats (*N* = 4 for each group) were obtained from the Aging Tissue Bank (Pusan National University, Republic of Korea). Young female rats were purchased from different sources: five 6‐week‐old rats from Orient Bio (Gyeonggi, Republic of Korea) and five 6‐month‐old rats from Central Lab. Animal Inc. (Seoul, Republic of Korea). All rats were intracardially perfused with saline and 10% neutral buffered formalin under deep anesthesia with isoflurane. The brains were then collected and fixed in 10% neutral buffered formalin for more than 3 days and then sealed in 15‐ml conical tubes for the MRI experiment. All experimental procedures were approved by the Institutional Animal Care and Use Committees (IACUC) of Ulsan National Institute of Science and Technology (UNISTIACUC‐19‐01).

### 2.2 Ex vivo MRI iron measurements in rat brains

The fixed rat brains were scanned with a 40 mm volume coil using a 7 T animal MRI scanner (7.0 T Bruker PharmaScan, Bruker Biospin) at the UNIST Central Research Facilities (Ulsan, Republic of Korea). The *T*_2_* maps were acquired using a multi‐gradient echo (MGE) sequence with repetition time (TR) = 4000 ms; echo time (TE) = 2.7, 6.1, …, and 50.3 ms (15 echoes); flip angle = 90°; matrix size = 256 × 256; field of view = 25 × 25 mm^2^; number of averages = 4; number of slices = 20; and slice thickness = 0.4 mm. Quantitative voxel‐wise *T*_2_* values were calculated by nonlinear least‐square curve fitting using the single exponential decay function S(TE) = S_0_ × e^−TE/T2*^, where S_0_ is the maximum signal intensity. The quantitative susceptibility mapping (QSM) algorithm reconstructed magnetic susceptibility images using the morphology‐enabled dipole inversion method (Liu et al., 2012), based on data obtained from MGE sequences. We obtained high signal‐to‐noise maps in the QSM process by reconstructing susceptibility images using only data from the three shortest TEs (2.7, 6.1, and 9.5 ms) in the MGE sequence. The mean of the whole brain was used as a reference for QSM quantification.

### 2.3 RNA sequencing of rat SN

After measuring brain iron content by ex vivo MRI, the *SN* was dissected from each scanned brain (Bregma: −5.5 mm) as described previously (Salvatore et al., 2012), and stored in the DNA/RNA shield (R1100; Zymo Research). For RNA extraction, the stored rat *SN* tissues were washed with cold PBS on an ice‐cold dish and then finely cut into several millimeter pieces with a razor blade. Next, total RNAs were extracted from collected slices using the PureLink FFPE RNA Isolation Kit (#K156002; ThermoFisher) following the manufacturer's instructions. The RNA quality was evaluated with BioAnalyzer RNA 6000 pico assay kit (5067–1513; Agilent Technologies). RNA‐seq libraries were next constructed using NEBNext Ultra Directional RNA Library Prep Kit for Illumina (E7420; New England Biolabs) according to the manufacturer's FFPE library preparation protocol, together with NEBNext Poly(A) mRNA Magnetic Isolation Module (E7490; New England Biolabs), and were sequenced using Illumina HiSeq 2500 with 150 PE configuration (Theragen Etex).

### 2.4 Cell culture

The human neuroblastoma cell line SH‐SY5Y was a kind gift from Prof. Chunghun Lim (UNIST). SH‐SY5Y cells were maintained in DMEM (#11965118; ThermoFisher) containing 10% FBS (#26140079; ThermoFisher) and 1% antibiotics (#15240062; ThermoFisher) and incubated under humidity at 37°C with 5% CO_2_. Iron cytotoxicity was evaluated by treating 1 × 10^4^ SH‐SY5Y cells with various concentrations of FeCl_2_ (5016–4405; Daejung Chemical & Metals) in a 96‐well plate for 24 h; a total of five replicates were included. After incubating the cells with WST‐8 Cell Viability Assay Kit, cell viability was quantified by measuring the absorbance at 450 nm for quantification and 690 nm as reference (QM2500; BioMax).

### 2.5 RNA‐seq of iron‐treated SH‐SY5Y cells

SH‐SY5Y cells (2.2 × 10^6^) were cultured on 100 mm dishes for 24 h. The medium was then replaced with either 1 or 2 mM FeCl_2_. Cells were passaged every 3–4 days by adding fresh FeCl_2_‐treated media. After 2 weeks, 0.3 × 10^6^ cells were seeded in three wells of a 6‐well plate; the medium was replaced with fresh FeCl_2_‐treated medium the next day. Non‐treated cells were prepared according to the same procedure of iron‐challenged cell culture but incubated in media without FeCl_2_. When untreated or iron‐treated cells reached 70~80% confluency in the 6‐well plates, the medium was aspirated, and lysis buffer was added to each well. RNA from each well was isolated using a Purelink RNA mini kit (12183018A; ThermoFisher) according to the manufacturer's protocol. RNA integrity was verified via an RNA 6000 pico assay run on Bioanalyzer, and mRNA was isolated using NEBNext Poly(A) mRNA Magnetic Isolation Module. From the mRNA, a transcriptome library was prepared using NEBNext Ultra II Directional RNA Library Prep Kit for Illumina following the manufacturer's protocol. After evaluation of the length of the library, the iron‐treated and untreated libraries were sequenced using Illumina NextSeq 500/550 with 150 cycles mid‐output kit (Cat # 20024904; Illumina).

### 2.6 RNA‐seq data analysis

Reads were processed with trimmomatic (version 0.39; Bolger et al., 2014) with the default option being the removal of adapter sequences and low‐quality reads. Filtered reads were mapped to the longest transcripts of each rat gene from Ensembl (version 98; Howe et al., 2021) using BWA‐MEM (version 0.7.17; Li, 2013). Differentially expressed genes were identified using DESeq2 (version 1.32.0; Love et al., 2014), with an adjusted *p*‐value less than 0.05 and absolute log2 fold‐change greater than 1.0. For rat brain transcriptome data, we considered genes with a read per kilobase per million (RPKM) greater than 1.0 among more than half of the total number of samples.

### 2.7 Pathway analysis

Differentially expressed genes were ranked based on their ‐log_10_(*p*‐value) multiplied with the sign of log_2_(fold‐change). Gene set enrichment analysis was performed using the PreRanked method with the Gene Set Enrichment Analysis (GSEA) software (version 4.1.0; Subramanian et al., 2005), and the enriched terms were filtered with a q‐value less than 0.05. Overrepresented gene ontology terms (biological processes) were analyzed with PANTHER (version 16.0; Mi et al., 2021). Gene ontologies of differentially expressed genes in each analysis were identified with all expressed genes as background using Fisher's exact test, and the false discovery rate (FDR) of less than 0.05 was applied to identify significantly enriched terms.

### 2.8 Identification of genes responding to iron stress

To determine candidate genes related to the iron stress response, we identified the overlap between differentially regulated genes in 15‐month‐old and 6‐month‐old rats (adjusted *p*‐value less than 0.05), and those in SH‐SY5Y cells treated with 2 mM FeCl_2_ versus controls with absolute log_2_ (fold‐change) greater than 1.0 and an adjusted *p*‐value less than 0.05 were selected.

### 2.9 Suppression of iron stress response genes

siRNA duplexes targeting CLU (5′‐AUG AUG AAG ACU CUG CUU‐3′; Zhang et al., 2014), HERPUD1 (5′‐GGA AGG CAC UGA UCC UGA AAC UGA AUU‐3′), and scramble (5’‐CCU CGU GCC GUU CCA UCA GGU AGU U‐3′) were purchased from Genolution. We validated their efficiency by transfecting them into 5 × 10^4^ SH‐SY5Y cells in a 24‐well plate using RNAiMAX (13,778,030, Thermo Fisher) following the manufacturer's protocol. RNA expression was quantified with quantitative real‐time PCR (qRT‐PCR) after 48 h and normalized to GAPDH expression. Using Purelink RNA mini kit, we purified total RNAs and synthesized cDNA with M‐MLV Reverse Transcriptase (RT001M; Enzynomics). The following primers were used for qRT‐PCR: CLU (forward, 5’‐TGC GGA TGA AGG ACC AGT GTG A‐3′ and reverse, 5′‐TTT CCT GGT CAA CCT CTC AGC G‐3′), HERPUD1 (forward, 5’‐CCA ATG TCT CAG GGA CTT GCT TC‐3′ and reverse, 5’‐CGA TTA GAA CCA GCA GGC TCC T‐3′) and GAPDH (forward, 5’‐GTC TCC TCT GAC TTC AAC AGC G‐3′ and reverse, 5’‐ACC ACC CTG TTG CTG TAG CCA A‐3′). To evaluate the role of these genes in the iron stress response, we transfected these siRNAs into 5 × 10^3^ SH‐SY5Y cells (*n* = 5 replicates); the cells were incubated for 48 h and then treated with 0.0, 2.0, or 5.0 mM FeCl_2_ for 24 h. Cell viability was, furthermore, evaluated with WST‐8, according to the manufacturer's protocol.

## 3 RESULTS

### 3.1 Aging‐associated iron accumulation occurs in the midbrain of 15‐month‐old rats

**FIGURE 1:**
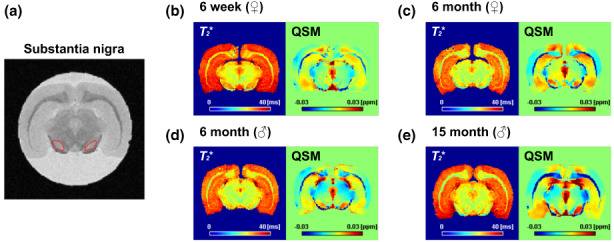
Representative magnetic resonance images of rat brains. (a) Proton density‐weighted image of rat midbrain. The *SN* region we investigated is highlighted in red. (b‐e) Four representative *T*_2_* maps and quantitative susceptibility mapping (QSM) results of the group of rat brains investigated in this study. (b) Six‐week‐old female rat, (c) 6‐month‐old female rat, (d) 6‐month‐old male rat, and (e) 15‐month‐old male rat. The QSM signal is increased (marked with the red color) in the *SN* in the older brain compared with the younger one.

Iron accumulation is associated with aging in various species (Han et al., 2021; Ripa et al., 2017). However, the time window of the age of animals that enables observation of iron accumulation that subsequently triggers the stress response in the brain needs to be determined. In the present study, we obtained 15‐month‐old and 6‐month‐old rats from the Aging Tissue Bank (Pusan National University, Busan, Republic of Korea). Next, we obtained relatively younger (6‐month‐old and 6‐week‐old) rats for a more comprehensive analysis and measured their brain iron accumulation with MRI. Since iron exhibits paramagnetic properties and hence generates susceptibility‐induced, non‐uniform magnetic field changes that affect MRI transverse relaxation parameters and phases (Haacke et al., 2005; Hametner et al., 2018), *T*_2_* and QSM are used for the quantitative analysis of iron content. Representative MRI data from each age group are shown in Figure 1. We focused on the *SN* (red lines in Figure 1a) because it contains high levels of iron in the aged human brain (Zecca et al., 2004). Representative MRI images showed a decrease in *T*_2_* and increased magnetic susceptibility values with aging in the *SN* (Figure 1b‐e).

**FIGURE 2:**
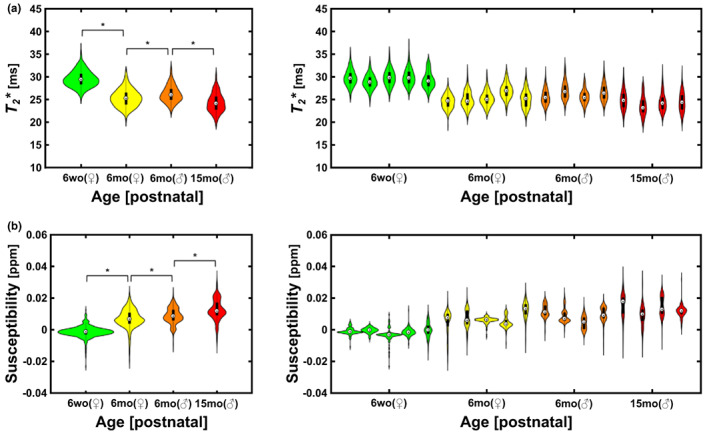
Summary of ex vivo *T*_2_* and quantitative susceptibility mapping (QSM) measurements. (a) *T*_2_* and (b) magnetic susceptibility were used to infer the iron accumulation of the *SN* of rat brains. Although there is individual variability in each age/sex group (right panel; green for 6‐week‐old female rat, yellow for 6‐month‐old female rat, orange for 6‐month‐old male rat, and red for 15‐month‐old male rat), we confirmed that the older group has significantly higher signals of iron accumulation (left panel; Wilcoxon rank‐sum test; *p* < 0.0001 is denoted as *).

In addition, we quantitatively analyzed the MRI data. The overall *T*_2_* and QSM data are shown in Figure 2. Mean *T*_2_* values significantly decreased with aging (*p* < 0.0001, Wilcoxon rank‐sum test), and mean magnetic susceptibility values increased with aging (*p* < 0.0001, Wilcoxon rank‐sum test; Figure 2). Moreover, it was confirmed that sex‐based differences were significant in the 6‐month‐old group (*p* < 0.0001, Wilcoxon rank‐sum test) for both mean *T*_2_* and magnetic susceptibility values. These trends were observed via the individual *T*_2_* and magnetic susceptibility values of each sample.

### 3.2 Transcriptome analysis reveals significant alterations in gene expression in the midbrain of 15‐month‐old rats

**FIGURE 3:**
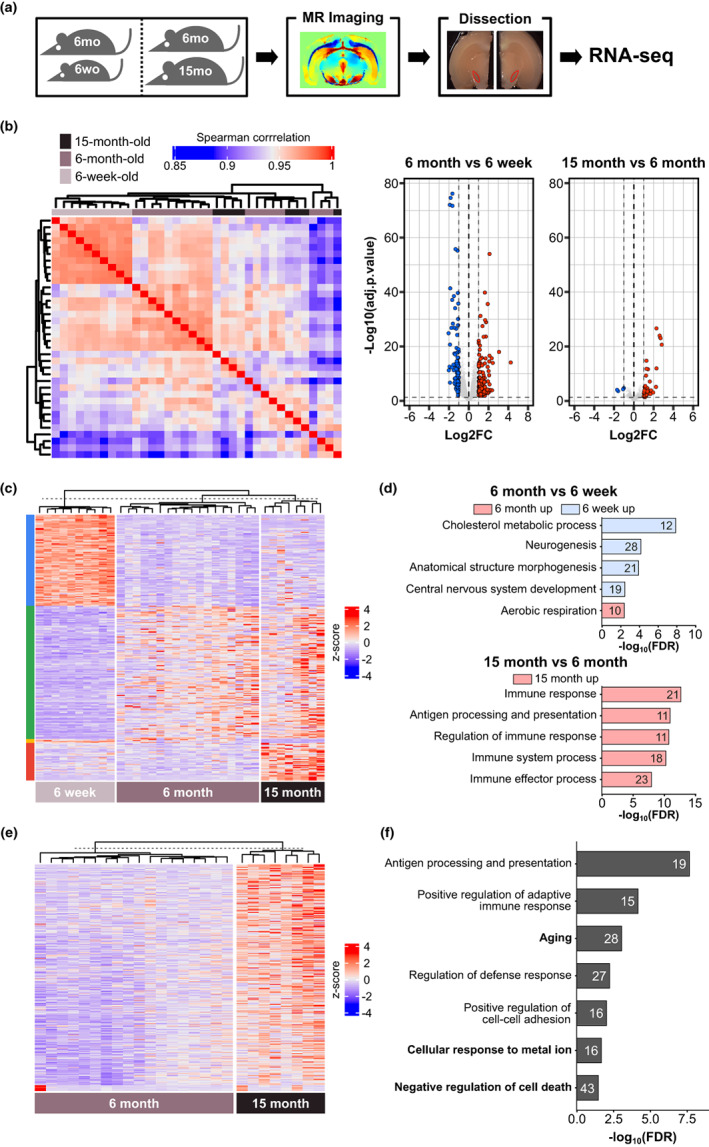
Gene expression profiles of *SN* dissected from magnetic resonance imaging analyzed rats. (a) Schematic diagram of experiment. (b) The correlation of expression profiles of individual rat *SN* tissues. Gene expressions of 6‐week‐old rats are highly correlated, but those of older rats (6‐month‐old and 15‐month‐old) were relatively discordant, possibly due to their genetic diversity. (c) Age‐dependent differentially expressed genes (DEGs). We identified DEGs with a greater than twofold difference in their normalized abundance and adjusted *p*‐value less than 0.05. We converted the RPKM values to the z‐score across samples for visualization in this heatmap. (d) Biological processes enriched in up‐ and downregulated genes in 6‐week‐old rats vs. 6‐month‐old rats (upper panel) and 6‐month‐old vs. 15‐month‐old rats (bottom panel). (e) DEGs in the *SN* of 15‐month‐old rats versus those of 6‐month‐old rats. (f) Biological processes enriched in upregulated genes in the *SN* of 15‐month‐old rats.

To examine responses in gene expression to iron accumulation in the *SN*, we dissected the region where the iron is accumulated based on our MRI analysis and performed RNA‐seq experiments on these samples (Figure 3a, Table S1). Our bulk RNA‐seq approach could not distinguish dopaminergic neurons in the *SN*; however, we expected that enrichment of those cells could be sufficient to capture cellular responses against iron overload. We only identified 43 differentially expressed genes in 15‐month‐ and 6‐month‐old rats (39 genes were upregulated in 15‐month‐old‐ and four in 6‐month‐old rats) (Table S3). On the contrary, 234 genes were identified in 6‐month‐ and 6‐week‐old rats (95 genes were upregulated and 139 genes were downregulated in 6‐week‐old rats versus 6‐month‐old rats) (Table S3). Based on a correlation of gene expression among biological replicates, the midbrains from 6‐week‐old rats showed similar gene expression profiles. However, midbrains from 6‐month‐old and 15‐month‐old rats were highly variable among individuals in the same group and between groups (Figure 3b,c). Therefore, we reasoned that the modest number of differentially expressed genes in 15‐month‐old versus 6‐month‐old rat midbrains was caused by the heterogeneity of older rat midbrain samples.

To identify cellular responses of the midbrain at different ages (and thereby different levels of iron overload), we, furthermore, analyzed gene ontology terms from differentially expressed genes. MRI analysis confirmed that iron was first detected in the *SN* 6 months after birth (Figure 1). Genes responding to iron overload may thus not be functional at this time point. As expected, most neuronal development‐related terms were enriched in 6‐week‐old rats. However, the immune response‐related term suggested in brain aging (Lucin & Wyss‐Coray, 2009) was mainly associated with genes upregulated in 15‐month‐old rats (Figure 3d). For the following analysis, we decide to focus on 15‐month‐old and 6‐month‐old rat data because we were interested in finding genes linked to the iron response, independent of neurodevelopment.

We attempted to find iron response‐related genes in aged rat data; however, but they were not satisfied with our stringent criteria to select differentially expressed genes, mostly having less than a twofold difference in their average expression level in aged rats compared with young ones. Therefore, we broadened the scope of differentially expressed genes without considering the fold‐change cutoff and discovered 355 genes that were upregulated in 15‐month‐old (Figure 3e). Pathway analysis confirmed that these genes were associated with immune response, aging, metal ion response, and apoptosis (Figure 3f). By contrast, our study found 309 downregulated genes in 15‐month‐old rats; however, we were unable to find any significant gene ontology terms. Taken together, we identified genes that were differentially expressed in the *SN* of 15‐month‐old rats; this included apoptosis‐related genes associated with iron accumulation‐mediated toxicity.

### 3.3 Genes associated with ER stress because of improper protein folding are actively expressed against iron overload in vitro

In this study, we compared gene expression in the *SN* from rats of different ages. It is, therefore, possible that several expressed genes may be related to brain development rather than to the cellular response to iron. To identify genes that respond specifically to iron overloading, we examined the expression of iron‐treated SH‐SY5Y neuroblastoma cells and further investigated the overlap of differentially expressed genes reported in our in vivo rat brain data.

**FIGURE 4:**
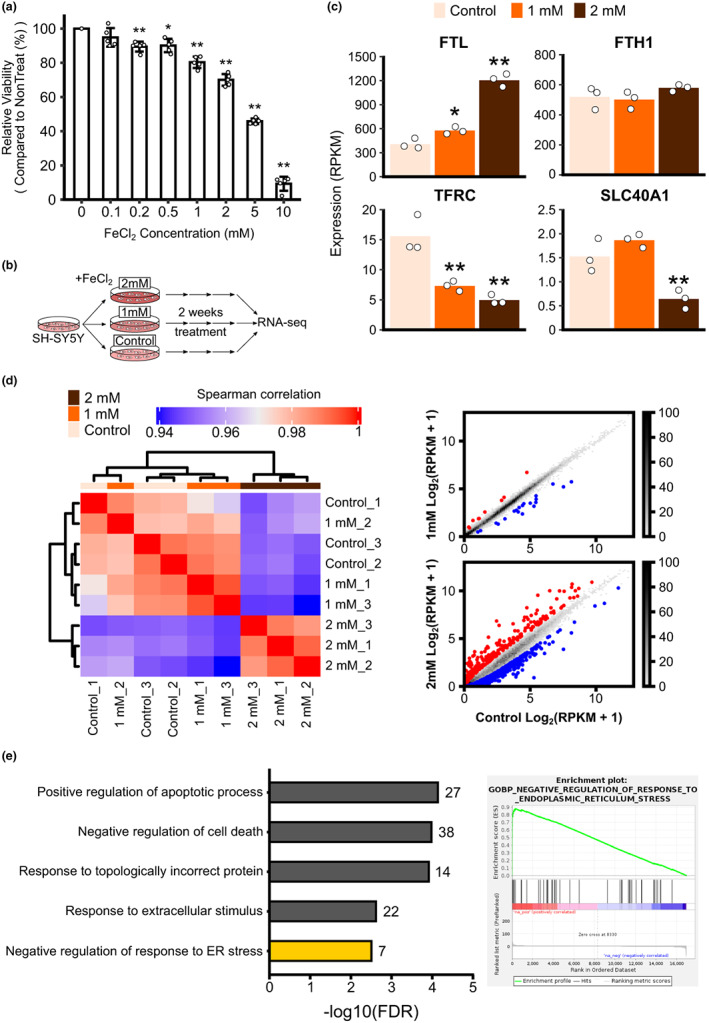
Differentially expressed genes in the iron‐exposed neuroblastoma cell. (a) The cell viability of neuroblastoma cell line SH‐SY5Y progressively decreases when the iron concentration is increased (*N* = 5). Compared with untreated controls, cells with more than 1 mM FeCl_2_ show significantly reduced cell viability, based on one‐way ANOVA followed by Tukey's post hoc test (*p*‐value less than 0.01, noted with **). (b) Schematic diagram of iron challenging experiment. (c) Genes related to iron homeostasis showed that 2 mM FeCl_2_ treatment could induce their gene expression changes by iron exposure (adjusted *p*‐value less than 0.05(*) or 0.01(**) from DESeq2 analysis). (d) The correlation of gene expression also showed that treatment with 2 mM FeCl_2_ altered gene expression, different from the untreated control. (e) Biological processes enriched in the deferentially expressed genes in treating with 2 mM FeCl_2_.

To find the optimal iron concentration for our experiment, we treated SH‐SY5Y cells with various concentrations of FeCl_2_ and recorded the cytotoxicity. Cell viability decreased significantly in the presence of 200 μM FeCl_2_; 49% of cells survived after 24 h of treatment with 5 mM FeCl_2_ (Figure 4a), but most cells died after 5 days of treatment at this same concentration. We, therefore, used 2 mM FeCl_2_ to mimic an iron overload state, while considering the iron content of a healthy nigrosome 1 region (Friedrich et al., 2021). To observe the progressive effect of iron concentration, we also prepared SH‐SY5Y cells treated with 1 mM FeCl_2_. However, we could observe 6 downregulated genes because of the high gene expression correlation with control (data not shown). In other words, a 24‐h iron treatment was insufficient to induce transcriptome profile changes. SH‐SY5Y cells were, therefore, chronically challenged with FeCl_2_ for 2 weeks; RNA sequencing was then performed on these cells to examine alterations to their transcriptome (Table S2).

To investigate the iron overload state, we examined how different exogenous iron concentrations affected iron homeostasis‐related gene expression in SH‐SY5Y cells. With increasing iron concentration, the expression of the iron storage‐related gene *FTL* and *FTH1* increased, and the expression of the iron uptake‐related gene *TFRC* decreased (Figure 4b). Although the expression of the iron export‐related gene *SLC40A1* (also known as *FPN1*) was altered after exposure to 2 mM FeCl_2_, we hypothesized that treating SH‐SY5Y cells for 2 weeks with 2 mM FeCl_2_ would mimic the iron overload condition.

To analyze the overall profile of genes responding to iron overload under different iron concentrations, we examined the gene expression correlation of our RNA‐seq data in vitro. Cells treated with 1 mM FeCl_2_ for 2 weeks showed similar gene expression patterns to untreated controls. Cells treated with 2 mM FeCl_2_, however, showed distinctive expression patterns (Figure 4c). Next, we compared gene expression between 2 mM FeCl_2_‐treated SH‐SY5Y cells and untreated controls and identified 277 upregulated and 651 downregulated genes in cells treated with 2 mM FeCl_2_ (Table S4).

To assess the enriched cellular processes in these differentially expressed genes, we analyzed the overrepresented gene ontology terms and confirmed that genes related to apoptosis and unfolded protein response in the ER stress were enriched in the iron overload conditions (Figure 4d). We, furthermore, confirmed the relationship between excessive iron accumulation and ER stress through GSEA (Figure 4d). These data suggest that iron overload induces ER stress via incorrect protein folding and eventual cell death in vivo.

### 3.4 HERPUD1 and CLU protect the SN from age‐related excessive iron accumulation‐induced toxicity

**FIGURE 5:**
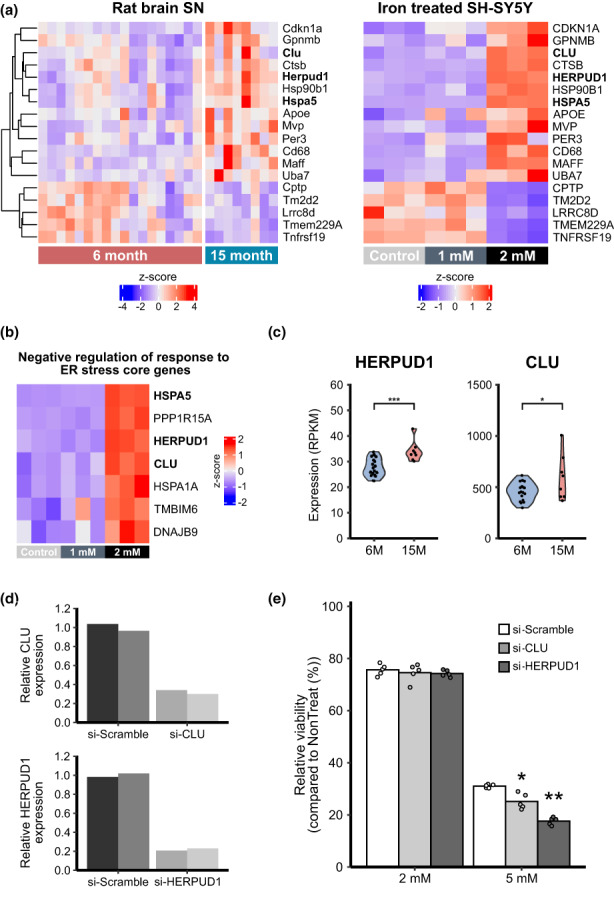
Gene expression changes in the *SN* of aged rats due to iron overload. (a) The overlap of significantly differentially expressed genes in the *SN* of 15‐month‐old rats versus those of 6‐month‐old rats, and SH‐SY5Y cells exposed to a high concentration of iron (2 mM FeCl_2_). (b) Three genes (HSPA5, HERPUD1, and CLU) related to “negative regulation of response to ER stress core genes” (GO:1903573) are highly expressed with respect to iron overload. (c) Among these genes, HERPUD1 and CLU are also upregulated in the 15‐month‐old rats compared with 6‐month‐old rats (adjusted *p*‐value less than 0.05(*) or 0.005(***) from DESeq2 analysis). (d) siRNAs reduced the expression of CLU and HERPUD1 to about 20% of the normal expression level. (e) When CLU and HERPUD1 gene expression were reduced, cell viability also decreased when exposed to iron, confirming their role in iron tolerance based on one‐way ANOVA followed by Tukey's post hoc test (*p*‐value less than 0.05 noted with (*) and less than 0.01, (**)).

We analyzed the overlap of differentially expressed genes in 15‐month‐old rat midbrains and in 2 mM FeCl_2_‐challenged SH‐SY5Y cells to identify genes related to iron responses based on our aged rat *SN* transcriptome data. We identified 13 upregulated genes (hereafter referred to as “iron‐rich genes”) and five downregulated genes under iron‐rich conditions (Figure 5a). We also found that incorrect protein folding‐related genes were highly enriched in 2 mM FeCl_2_‐challenged SH‐SY5Y cells. Therefore, we tested whether these iron‐rich genes were strongly associated with features of ER stress. We revisited the GSEA data with 2 mM FeCl_2_ and found that iron‐rich genes were included in the lists of core genes of the “Negative regulation of response to ER stress” term. Among the seven core genes upregulated in cells challenged with 2 mM FeCl_2_ (Figure 5b), only three genes (HSPA5, CLU, and HERPUD1) were found in iron‐rich genes and their expressions were significantly upregulated in 15‐month‐old compared with 6‐month‐old rats (Figure 5c).

The heat‐shock 70 kDa protein 5 (HSPA5, also known as BiP) protects the ER from unfolded protein stress as a molecular chaperone (Zhu et al., 2017). HSPA5 is already known to antagonize the ferroptosis (Zhu et al., 2017). Therefore, we investigated the role of the other two genes (CLU and HERPUD1) in response to an iron overload state. We suppressed CLU and HERPUD1 expression with validated siRNAs (Figure 5d) and measured the cell viability in the presence of various concentrations of iron. With the treatment of 5 mM FeCl_2_ for 24 h, cell viability was reduced after CLU knockdown; cell viability also decreased in cells in which HERPUD1 expression was knocked down (Figure 5e). Overall, our data suggest that CLU and HERPUD1 are involved in the cellular response to age‐related iron overload in the *SN* and may protect cells from iron‐induced ER stress.

## 4 DISCUSSION

During healthy aging, iron accumulates in the *SN*, as confirmed by neutron activation analysis and Perl's staining with postmortem samples (Zecca et al., 2004), and MRI in healthy subjects (Aquino et al., 2009; Möller et al., 2019). Also, iron storage compounds such as ferritin, neuromelanin (NM), and hemosiderin increase with age (Möller et al., 2019). Especially, NM may protect *SN* dopaminergic neurons by sequestering iron and neurotoxic dopamine in the cytosol (Zucca et al., 2017). Aging is a risk factor in neurodegenerative diseases, and age‐related iron deposition may play a critical role in disease progression. During iron overload conditions, activated microglia could phagocytose and degrade NM released from dying neurons (Zucca et al., 2017). As a result, redox‐active metals and toxic molecules liberated from NM may induce microglial activation and neuronal damage (Zucca et al., 2017). Moreover, hemosiderin which stores iron during iron burden releases iron more easily than ferritins and this process may be implicated in neurodegenerative disease (Möller et al., 2019). In the *SN*, excessive iron accumulation may contribute to PD pathogenesis, including alpha‐synuclein oligomerization and the loss of dopamine neurons. In aged mice, knocking out Nrf2 ameliorated brain iron deposition by preventing iron influx (Han et al., 2021). The molecular mechanisms that counter age‐related iron tolerance are still elusive. Concerning iron deposition, the present study analyzed gene expression profiles in the *SN* of rat brains to better understand gene responses to iron overload.

Magnetic resonance imaging (MRI) is a non‐invasive method that can be used to quantify iron accumulation; this allowed us to analyze the transcriptome of the same samples. We quantified iron levels in different ages of the rat *SN* with MRI. In agreement with previous studies (Han et al., 2021), we found high iron levels in the *SN* of old rats than in young rats. The transcriptome profiles of the *SN* in 6‐month‐old and 15‐month‐old rats, however, are not significantly distinguishable; this is mainly caused by their variability, possibly from different modulation ranges in age‐related pathways (Cellerino & Ori, 2017). Iron overload induces oxidative stress responses and is also known as a hallmark of aging (López‐Otín et al., 2013), and inflammation‐related genes are generally enriched in older animals (Lucin & Wyss‐Coray, 2009; Ximerakis et al., 2019). In the current study, we also observed that genes related to these pathways were strongly upregulated in 15‐month‐old rats.

To distinguish genes responding to iron accumulation from aging‐associated genetic features in our rat *SN* data and to identify the molecular responses under iron deposition, we analyzed global gene expression profiles of iron‐treated SH‐SY5Y cells. Acute iron treatment for 24 h upregulates an apoptotic pathway and proinflammatory cytokine‐related pathway (Bautista et al., 2016). Although we observed substantial cell death due to iron toxicity when we evaluated this acute iron overload condition, gene expression patterns between control and high‐dosage iron treatment did not differ significantly when we analyzed enriched functions relevant to the iron response. In the model of chronic iron stress during aging, we treated SH‐SY5Y cells with 2 mM FeCl_2_, similar to a quantity of iron in the aged, healthy, *SN* (Friedrich et al., 2021). Cells were cultured for 2 weeks with an iron‐treated medium. The expression of iron homeostasis‐related genes was regulated by increasing the iron concentration. Moreover, apoptosis‐related pathways were more highly enriched in FeCl_2_‐challenged cells than in untreated controls.

To determine genes related to the iron response from our aged rat *SN* gene expression data, we then analyzed differentially expressed genes that were concordantly regulated in the 15‐month‐old rat *SN* and 2 mM FeCl_2_‐challenged cells. Under iron‐rich conditions, we identified 13 upregulated genes and five downregulated genes. Based on the premise that maintaining proteostasis during stress or resting conditions is compromised by aging (Labbadia & Morimoto, 2015), as shown in aged rats (Paz Gavilán et al., 2006), we hypothesized that the expression of ER stress‐related genes that are upregulated in iron‐treated cells would also increase in the *SN* of aged rats. We thus identified genes that strongly contribute to the negative regulation of ER stress pathway enrichment in iron‐challenged cells with the gene set enrichment analysis and asked whether they are also overexpressed in the 15‐month‐old rat *SN*. *HSPA5*, *HERPUD1*, and *CLU* expression increased in vivo and cultured neuroblastoma cells. Following 2 weeks of challenging microglia with iron, iron overload promotes ER stress by increasing oxidative damage (Angelova & Brown, 2018). Moreover, in iron‐loaded astrocytomas, ER chaperones are upregulated, suggesting that a high dose of iron may induce protein misfolding (Lovejoy & Guillemin, 2014). Our cell culture study also showed that incorrect protein folding‐related and ER stress‐related functions were activated in iron‐challenged SH‐SY5Y cells. These data suggest that aging‐related iron overload chronically induces ER stress via incorrect protein folding.

From the three genes that overlapped between our in vivo and in vitro gene expression analysis, we focused on HERPUD1 and CLU. The chaperone HSPA5 is involved in ferroptosis (Zhu et al., 2017) and contributes to an iron‐dependent non‐apoptotic cell death that stabilizes GPx4 and indirectly attenuates lipid peroxidation in glioma cells (Chen et al., 2019); therefore, we excluded HSPA5 from further study. Homocysteine‐induced ER stress‐inducible ubiquitin‐like domain member 1 (HERPUD1), or Herp, is involved in the response to unfolded proteins. HERPUD1 is highly expressed following ER stress; it is, therefore, possible to protect neurons against induced ER stress by maintaining ER Ca^2+^ homeostasis and inhibiting caspase‐3 activation (Chan et al., 2004). HERPUD1 knockdown in SH‐SY5Y cells induces caspase‐3 activation and apoptosis after hemin treatment (Ding et al., 2017). Clusterin (CLU) has been investigated for its relationship to AD because of its activity as a chaperone by clearing misfolded proteins such as amyloid‐beta (Narayan et al., 2012). CLU retro‐translocates from the ER to the cytoplasm under conditions of stress to inhibit apoptosis by reducing protein aggregation (Zhang et al., 2014). Neuroblastoma cells treated with a sub‐lethal level of iron exhibit increased clusterin protein and expression levels (Strocchi et al., 2006). These data suggest that HERPUD1 and CLU may protect neurons from iron‐induced ER stress.

In the present study, we showed that CLU and HERPUD1 are important in the aged *SN* by counteracting iron accumulation via gene‐knockdown in iron‐challenged SH‐SY5Y cells. Exposure of cells to 2 mM FeCl_2_ did not alter cell viability; however, cell viability sharply decreased in the presence of 5 mM FeCl_2_. Downregulation of CLU or HERPUD1 would increase the sensitivity of iron‐induced ER stress; however, our data suggest that treatment with 2 mM FeCl_2_ is insufficient for generating a stress response. Moreover, the viability of iron‐challenged cells during chronic treatment with 2 mM FeCl_2_ supports this result. However, exposure to 5 mM iron, a similar concentration found in the *SN* of patients with PD (Friedrich et al., 2021), would cause iron‐induced stress that may overwhelm the protein misfolding stress‐buffering mechanism, promoting cell death. The components that participate in this pathway will need to be elucidated.

Our in vivo transcriptome data were based on dissected tissues. In comparison with published single‐cell transcriptome data, we could not identify which cell types were primarily responsible for the iron overload. We, therefore, further explored the expression of HERPUD1 and CLU in the published single‐cell transcriptome dataset. In the aged mouse brain, HERPUD1 is overexpressed throughout the entire brain including in microglia; CLU, however, was downregulated in most cell types, including some astrocyte subtypes (Ximerakis et al., 2019). In another data, however, CLU is highly expressed in astrocytes than in other cell types in the *SN* (Agarwal et al., 2020). The inconsistency of the CLU expression pattern may be explained by the heterogeneity of neuronal subtypes across brain regions during aging (Batiuk et al., 2020), or local transcriptional responses to age‐related iron accumulation in the *SN*, which could be identified by spatially analyzing gene expression profiles (Rodriques et al., 2019). To elucidate the in vivo cellular responses against iron accumulation at the single‐cell level, we require an improved method to measure iron levels at a similar resolution; however, this is still difficult to achieve using MRI.

In conclusion, by investigating the response of neurons in the *SN* against age‐related iron accumulation, we identified a transcriptome profile of aging‐related iron accumulation using rats of different ages and confirmed their iron accumulation using the MR images. To identify iron response‐related genes from mixed aging‐related genetic features, we analyzed gene expression of SH‐SY5Y cells chronically challenged with iron and observed that incorrect protein folding‐related ER stress was associated with iron accumulation both in vivo and in vitro. Moreover, two genes, CLU and HERPUD1, responded to age‐related iron accumulation, and the knockdown of these genes severely impaired the cellular tolerance for iron toxicity. We conjecture that the understanding of the gene expression landscape during age‐related iron accumulation can help us to elucidate molecular pathways and putative preventative strategies against neurodegenerative diseases.

## AUTHOR CONTRIBUTIONS

KK, HC, HJC, and TK conceived and designed the project. KK performed the transcriptome experiments and the cell‐based validation experiments, and HC prepared the animal and did the MRI analysis. KK, SL, EJC, WY, and CYLK performed the additional animal experiments with primary tissues under the supervision with HSJ, J‐IK, and TK. All authors analyzed the data and wrote the manuscript together. All authors also approved the final version.

## FUNDING INFORMATION

Korea Health Industry Development Institute, Grant/Award Number: HI18C0713; National Research Foundation of Korea, Grant/Award Number: 2017H1A2A1046162, 2018R1A6A1A03025810 and 2021R1A4A1031644; Ministry of Health Singapore, Grant/Award Number: MOE‐T2EP30121‐0032; National Medical Research Council, Ministry of Health‐Singapore, Grant/Award Number: NMRC‐OFIRG21jun‐0037; National Research Foundation Singapore, Grant/Award Number: NRF‐CRP17‐2017‐04; Ulsan National Institute of Science and Technology, Grant/Award Number: 1.220023.01

## CONFLICT OF INTEREST

The authors declare that they have no competing interests.

## Supporting information


Table S1. Gene expression table of rat *substantia nigra*.

Table S2. Differentially expressed gene analysis result table of rat *substantia nigra*.

Table S3. Gene expression table for iron‐exposed SH‐SY5Y.

Table S4. Differentially expressed gene analysis result table of iron exposed SH‐SY5Y.

## Data Availability

The datasets generated for this study can be found in the ENA under accession PRJEB49065.
